# Association between illegal drug use and cigarette smoking among Ethiopian students: A systematic review and meta-analysis

**DOI:** 10.1371/journal.pone.0304948

**Published:** 2024-06-20

**Authors:** Chala Daba, Mesfin Gebrehiwot, Sisay Abebe Debela, Belay Desye, Yonatal Mesfin Tefera

**Affiliations:** 1 Department of Environmental Health, College of Medicine and Health Sciences, Wollo University, Dessie, Ethiopia; 2 Department of Public Health, College of Health Science, Salale University, Fitche, Ethiopia; 3 Department of Public Health, College of Medicine and Health Sciences, Adigrat University, Adigrat, Ethiopia; 4 Adelaide Exposure Science and Health, School of Public Health, University of Adelaide, Adelaide, Australia; Bahir Dar University, ETHIOPIA

## Abstract

**Introduction:**

Cigarette smoking is a persistent public health problem as it is a risk factor for many diseases. Previous studies on the role of illegal drug use in cigarette smoking have yielded disparate and inconclusive results, hindering the development of effective intervention strategies to address this issue. Therefore, this systematic review and meta-analysis aimed to estimate the pooled prevalence of cigarette smoking and its associated factors, with a specific focus on the influence of illegal drug use among students in Ethiopia.

**Methods:**

We conducted a comprehensive search of international databases, including PubMed, Cochrane Library, Science Direct, CINAHL, African Journals Online, HINARI, Global Health, and Google and Google Scholar. Grey literature was also identified from various university digital libraries. The study followed the Preferred Reporting Items for Systematic Reviews and Meta-Analysis Protocols (PRISMA) guidelines. Due to the high heterogeneity among the included studies (I^2^ = 98.6%; p-value <0.001), we employed a random-effects model with a 95% confidence interval (CI) to estimate the pooled effect using STATA 14 software. The publication bias was assessed using a statistical Egger regression test.

**Results:**

A total of 22 studies involving 18,144 students met the eligibility criteria for this systematic review and meta-analysis. The pooled prevalence of lifetime and current cigarette smoking among students in Ethiopia was 13.8% (95% CI: 9.90–17.82) and 9.61% (95% CI: 7.19–12.03), respectively. Students who used illegal drugs were twenty-three times more likely to smoke cigarettes compared to their counterparts (OR = 23.57, 95% CI: 10.87–51.1). Living in urban settings (OR = 2.9; 95% CI: 1.15–7.28) and the habit of alcohol consumption (OR = 4.79; 95% CI: 1.57–14.64) were also identified as factors associated with cigarette smoking.

**Conclusions:**

We found that more than one in eight students in Ethiopia have engaged in lifetime cigarette smoking. Notably, students who used illegal drugs exhibited a significantly higher likelihood of cigarette smoking. In light of these findings, it is imperative to implement comprehensive public health interventions that target illegal drug use, cigarette smoking, and alcohol consumption, with a particular emphasis on urban residents.

## Introduction

One-third of the global population consumes tobacco products, including cigarette [1]. In 2019, as per the World Health Organisation (WHO) report, over 24 million individuals between the ages of 13 and 15 engaged in cigarette smoking [2]. In 2016, an estimated 13.8 million students between the ages of 15 and 16 used illegal drugs, with the highest prevalence among students in Europe [3, 4].

In developing countries, cigarette smoking is a neglected health problem and a significant cause of adult mortality and morbidity [5], with 80% of tobacco-related mortality and morbidity occurring in these regions [6]. Evidence from a systematic review and meta-analysis in Sub-Saharan Africa showed that the magnitude of substance abuse, including cigarette smoking and illegal drug use, was 41.6% [7]. It is also well documented that cigarette smoking is a significance risk factors for the majority of psychiatric disorders [8, 9]. Evidence from meta-analysis showed that cigarette smoking could increase the risk of dementia and the development of Alzheimer disease [10]. Likewise, observational studies linked cigarette smoking with increased risk of a number of psychiatric disorders, including suicide, major depressive disorder, and bipolar disorder [11, 12]. Beyond its health implications, cigarette smoking also has a detrimental impact on academic performance, increasing the risk of unwanted pregnancies, and unprotected sexual activity [4, 7, 13–15].

In Ethiopia, there is a growing and persistent issue of cigarette smoking among students, both at Universities and high schools, posing a significant public health concern [16]. This risk is exacerbating mainly due to high prevalence of illegal drug use among students. Notable statistics include a 43.5% prevalence of cigarette smoking among Dire-Dawa University students [17], 39.5% among Haramaya University students [18], and 28.6% among high school students in Oromia and the Southern Nation, Nationality and People Region (SNNPR) [19].

The previous studies conducted on the link between illegal drug use and cigarette smoking among high school and university students were highly dispersed and inconclusive [20–23]. While there is an acknowledgement of an increasing prevalence of cigarette smoking and its impact on public health, there is a dearth of a consolidated analysis that integrates data on illegal drug use and cigarette smoking, particularly concerning students. Therefore, this systematic review aims to address this gap by providing a thorough examination of the collective prevalence of cigarette smoking and its determinants among Ethiopian students, with special attention to the role of illegal drug use. The findings of this systematic review can be instrumental in informing evidence-based public health interventions and policies aimed at tackling the growing issue of cigarette smoking among Ethiopian students.

## Methods and materials

**Protocol registration:** This systematic review has been registered in the International Prospective Registry of Systematic Review (PROSPERO) with a specific registration number CRD42023443461.

### Study selection, search strategy and study period

This systematic review and meta-analysis followed the Preferred Reporting Items for Systematic Reviews and Meta-Analysis Protocols (PRISMA) guidelines [24] (S1 Checklist). The studies were retrieved from international electronic databases; PubMed, Cochrane Library, Science direct, CINAHL, African Journals Online, HINARI, Global Health, and Google and Google Scholar searches. Grey literature was also identified from different universities digital libraries. The following key terms were used to search the studies: "impact", "effect", "illegal drug use", "illicit drug use", "cigarette smoking", "tobacco smoking", "tobacco consumption", "tobacco chewing", "university student", "student", "high school student", "college student", "associated factors", "risk factors", and "Ethiopia". All key terms were combined using the Boolean operators “AND” or “OR” as appropriate. The search was carried out from July 1 to August 15, 2023, by four authors independently (CD, MG, BD, and SAD). Those studies searched from selected databases were transferred to Endnote, and duplicate files were excluded.

### Inclusion and exclusion criteria

In this meta-analysis, we included observational studies (cross-sectional, case-control, and cohort studies) on cigarette smoking and associated factors among students in Ethiopia. However, qualitative studies, unretrievable studies, editorial letters, studies with poor methodological quality, and studies that did not report the outcome of interest were excluded from the meta-analysis.

### Outcome assessment

The primary aim of this study was to determine the prevalence of cigarette smoking among Ethiopian students. Besides, the study aimed to identify the factors associated with lifetime cigarette smoking in the form of a log odds ratio.

### Data extraction and risk of bias assessment

Two authors (CD and MG) independently extracted the necessary data using Microsoft Excel 2013. The data extraction template consisted of various study details, such as author names, region and type of student (S1 File). After removing duplicates, two authors (CD and SAD) screened the relevant articles for inclusion. The quality of each article was evaluated using the Joana Brigg Institute (JBI) critical appraisal checklist [25] (S2 File). Each study’s quality was independently assessed on a scale of 100% by the five authors (CD, BD, MG, YM, and SAD). In cases of any discrepancies during the quality assessment, the mean score was calculated from the results of all reviewers to resolve differences.

### Statistical analysis

STATA version 14 software was used to analyse the data. A Forest plot was used to present the prevalence of lifetime and current cigarette smoking among students in Ethiopia. Because extreme heterogeneity was observed among the included studies (I^2^ = 98.6%, p-value <0.001), random-effects model was used to determine the pooled prevalence of cigarette smoking among students in Ethiopia. The random-effects model was used to determine the pooled prevalence of both lifetime and current cigarette smoking. Heterogeneity was assessed using the Higgs I^2^ statistic, with values of 25%, 50%, and, 75% indicating low, moderate, and high heterogeneity, respectively [26]. A p -value of less than 0.05 was considered indicative of the presence of heterogeneity.

A sensitivity analysis was performed to assess the influence of a single study on the pooled prevalence estimates. Subgroup analysis was also conducted based on various study characteristics, such as region (Oromo, SNNPR, Amhara, Tigray, or other), type of student (university, college, or high school). In addition, publication bias was assessed using Egger’s test with a p-value less than 0.05 suggesting a publication bias [27]. Moreover, univariable meta-regression analysis was conducted considering variables, such as type of student, region, and year of publication, in relation to the outcome variable.

## Results

### Study selection

**Fig 1:**
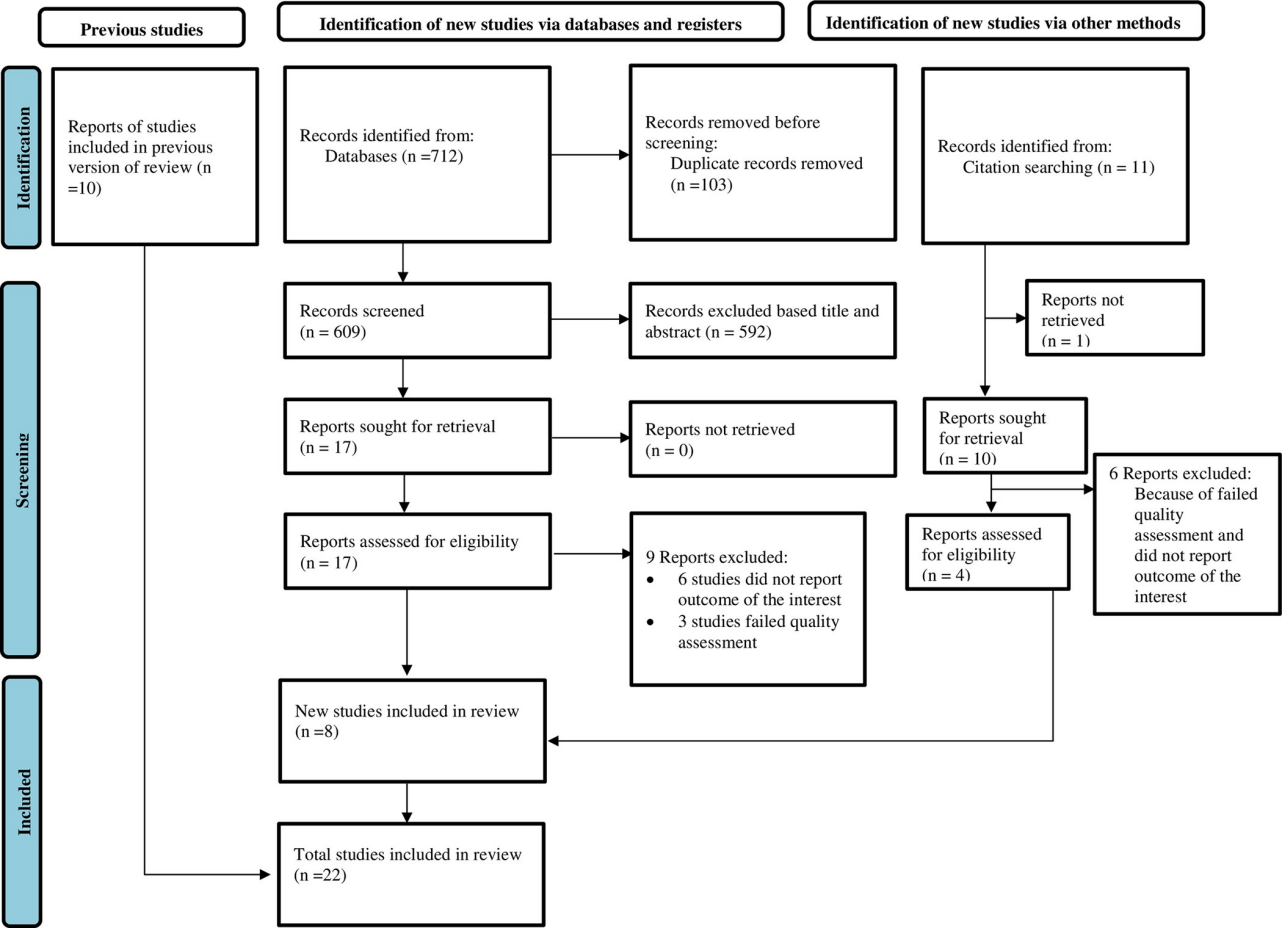
PRISMA flow diagram of the included studies for the systematic review and meta-analysis on the association between illegal drug use and cigarette smoking among students in Ethiopia, 2023.

A total of 712 articles were initially identified from database searches. Using the Endnote reference manager, 103 duplicate articles were removed; while 592 were excluded as they did not meet the inclusion criteria based on their titles and abstracts. Besides, 9 articles were excluded based on the quality of the assessment and the outcomes of the studies. Finally, 22 full-text articles were deemed eligible for systematic review and meta-analysis (Fig 1).

### Characteristics of the included studies

These studies involved a total of 18,144 participants, with fourteen studies focusing on university students [17, 18, 20, 22, 28–37], six studies on high school students [19, 21, 23, 38–40], and two studies on college students [41, 42]. Among the included studies, the highest reported prevalence of lifetime cigarette smoking was found in a study conducted in the Oromia region (39.5%) [18], while the lowest prevalence was observed in a study encompassing eleven regions of Ethiopia (4.7%) [23]. Regarding sample size, the studies included in the analysis ranged from a maximum of 3,457 participants [23] to a minimum of 188 participants [30] (Table 1).

**Table 1:** A descriptive summary of twenty-two studies included estimating the pooled prevalence of tobacco smoking among students in Ethiopia, 2023.

Authors	Year of publication	Region	Sample size (respond the question)	Response rate (%)	Lifetime prevalence (%)	Current prevalence (%)	Quality score (8)
Hagos et al [34]	2016	Tigray	271	100	11.4	5.0	7
Telayneh et al [42]	2021	Amhara	605	96.7	NA	6.8	6
Seid et al [40]	2021	Addis Ababa	383	97.7	9.6	6.4	7
Reda et al [39]	2012	Harari	1890	91.1	12.2	4.2	6
Dereje et al [19]	2014	SNNPR and Oromia	1704	98.2	28.6	17.2	7
Eticha et al [22]	2014	Tigray	193	100	NA	29.5	7
Deressa et al [30]	2011	Addis Ababa	632	98.4	9	1.8	8
Alebachew et al [18]	2019	Oromia	254	98.8	39.5	37.4	8
Hirpha et al [23]	2023	All regions	3457	97	4.7	2.4	7
Gebreslassie et al [33]	2013	Tigray	764	98.7	9.5	9.3	7
Gebremariam et al [32]	2018	Amhara	659	89	7.4	3.1	6
Tesfaye et al [36]	2014	Oromia	1040	98.3	22	10.8	6
Adere et al [28]	2017	Amhara	730	89.7	7.9	6.4	7
Bago et al [29]	2017	SNNPR	336	92.3	20.6	NA	8
Kumesa et al [41]	2020	Oromia	356	97.7	18.4	14.9	7
Banti et al [20]	2017	Somalia	648	92.3	NA	14.5	7
Tsegay et al [37]	2014	Amhara	845	94.6	11.3	3.9	6
Kumburi et al [17]	2017	Dire-Dawa	1239	75.1	43.5	41.2	6
Mekonen et al [35]	2017	SNNPR	747	97.1	5.7	NA	7
Dida et al [38]	2014	Oromia	603	97.9	13.1	4.6	7
Desta et al [31]	2018	Oromia	188	98.9	5.9	5.4	7
Duko et al [21]	2019	SNNPR	600	94	11	9.4	8

Hint: NA- Not available

### Meta-analysis

**Fig 2:**
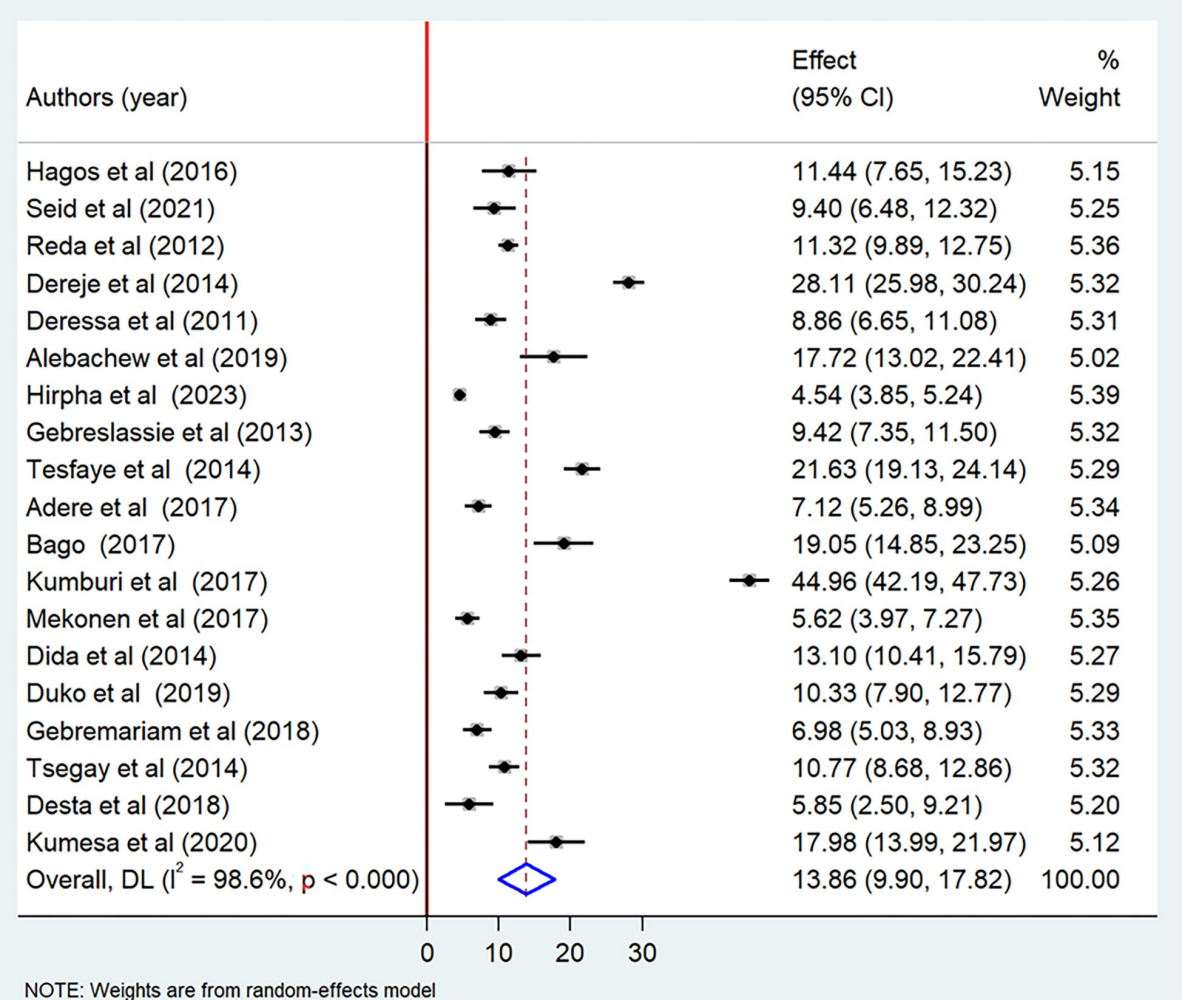
Forest plot of the pooled prevalence of lifetime cigarette smoking among students in Ethiopia, 2023.

**Fig 3:**
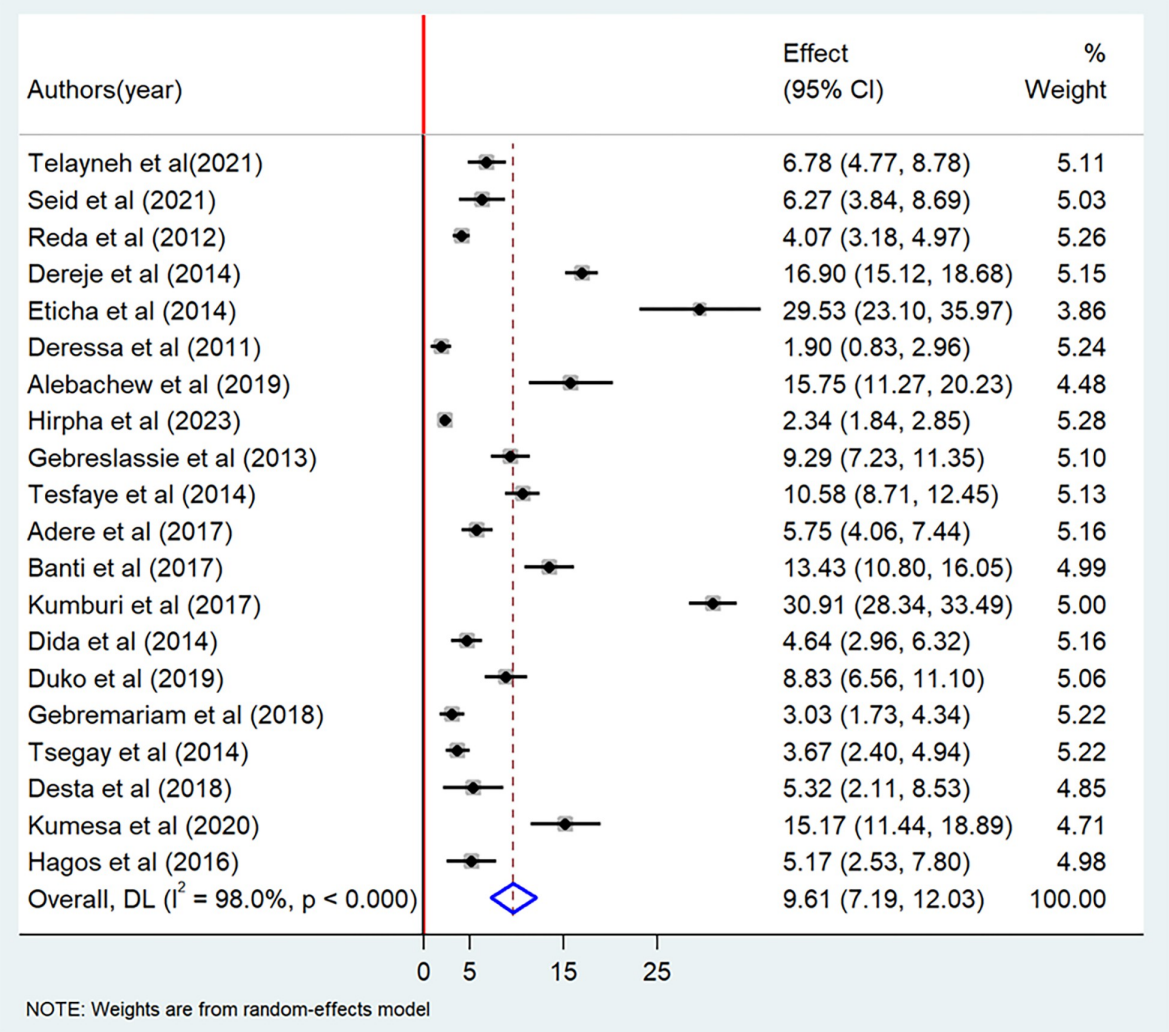
Forest plot of the pooled prevalence of current cigarette smoking among students in Ethiopia, 2023.

The current meta-analysis showed that the pooled prevalence of lifetime cigarette smoking among students in Ethiopia was 13.8% (95% CI: 9.90–17.82) (Fig 2). The pooled prevalence of current cigarette smoking among students in Ethiopia was 9.61% (95% CI: 7.19–12.03) (Fig 3).

### Test for publication bias

**Fig 4:**
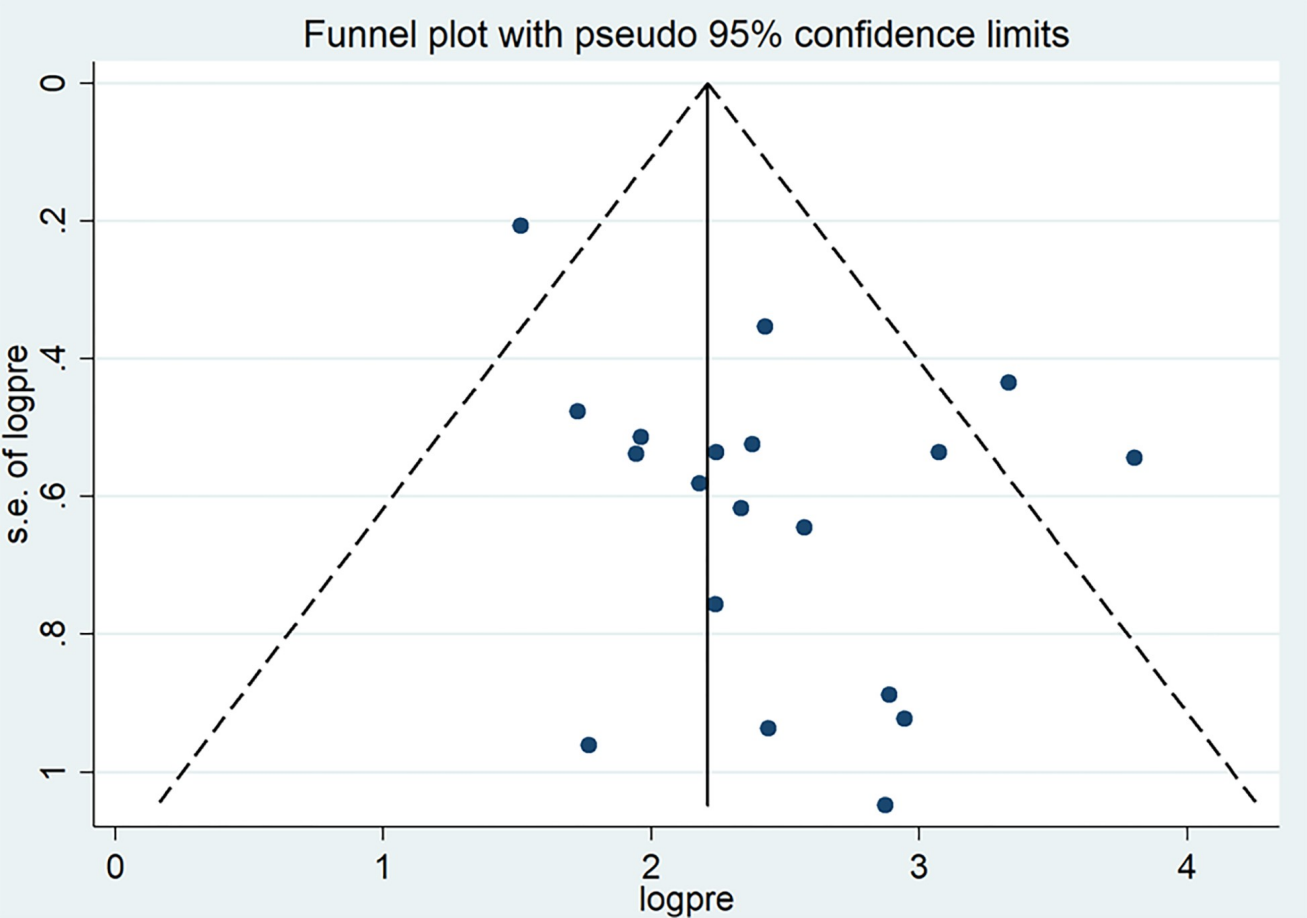
Funnel plot of the pooled prevalence of lifetime cigarette among students in Ethiopia, 2023.

**Fig 5:**
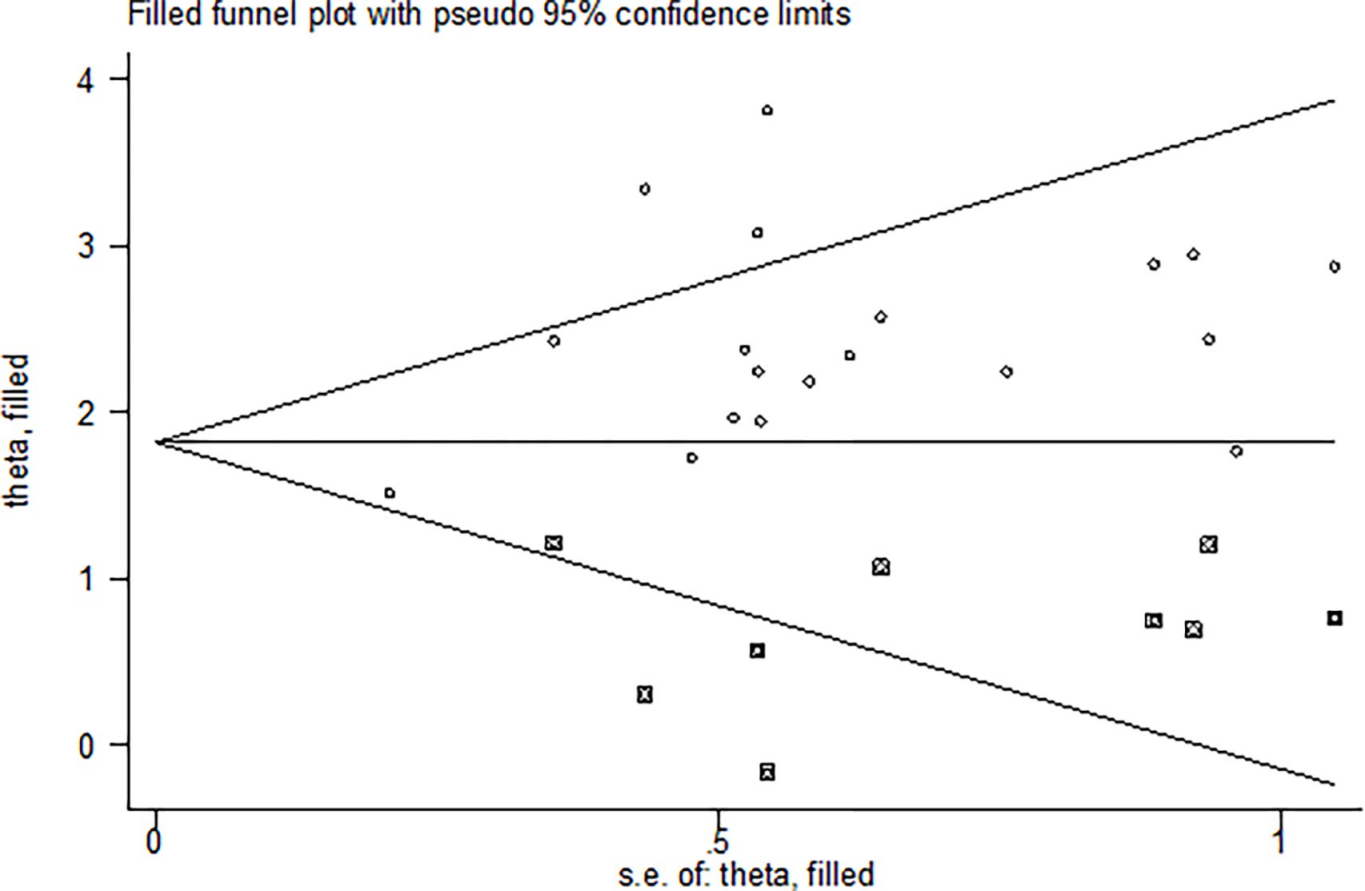
The funnel plot of a simulated meta-analysis.

Publication bias was assessed through a visual inspection of the funnel plot, revealing an asymmetric distribution that strongly indicated the presence of publication bias (Fig 4). Further statistical analysis employing the Egger regression test corroborated the significant presence of publication bias (p = 0.021). To pinpoint the sources of this bias, a trim and fill analysis was conducted, revealing notable variation in the newly estimated pooled odds ratio, denoted as the adjusted point estimate [OR = 1.89, (95% CI: 1.52–2.26)], when compared to the initial or observed point estimate [OR = 2.39, (95% CI: 2.05–2.73)] (Fig 5).

### Subgroup analysis

**Fig 6:**
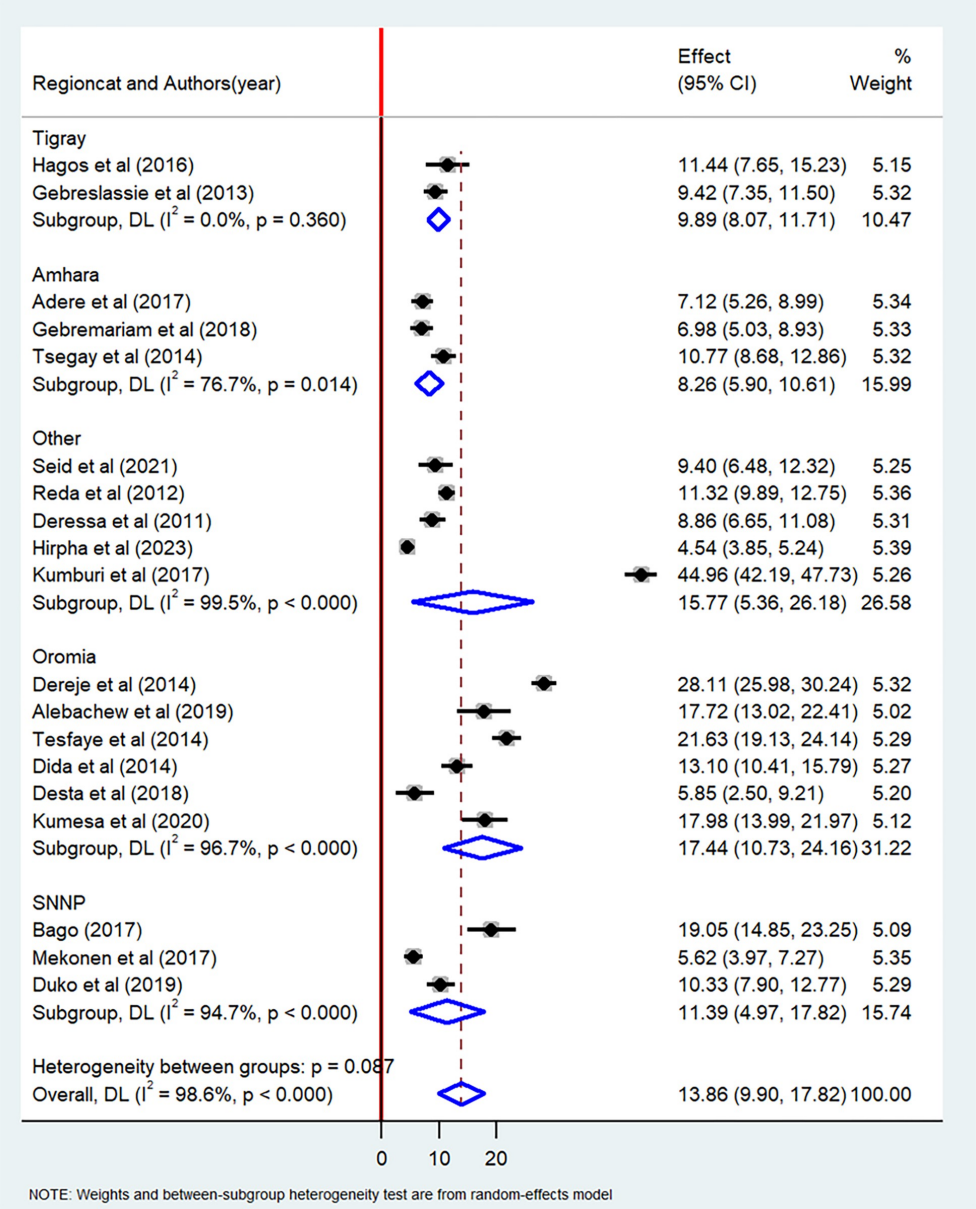
Subgroup analysis by region of the pooled prevalence of lifetime cigarette smoking among students in Ethiopia, 2023.

**Fig 7:**
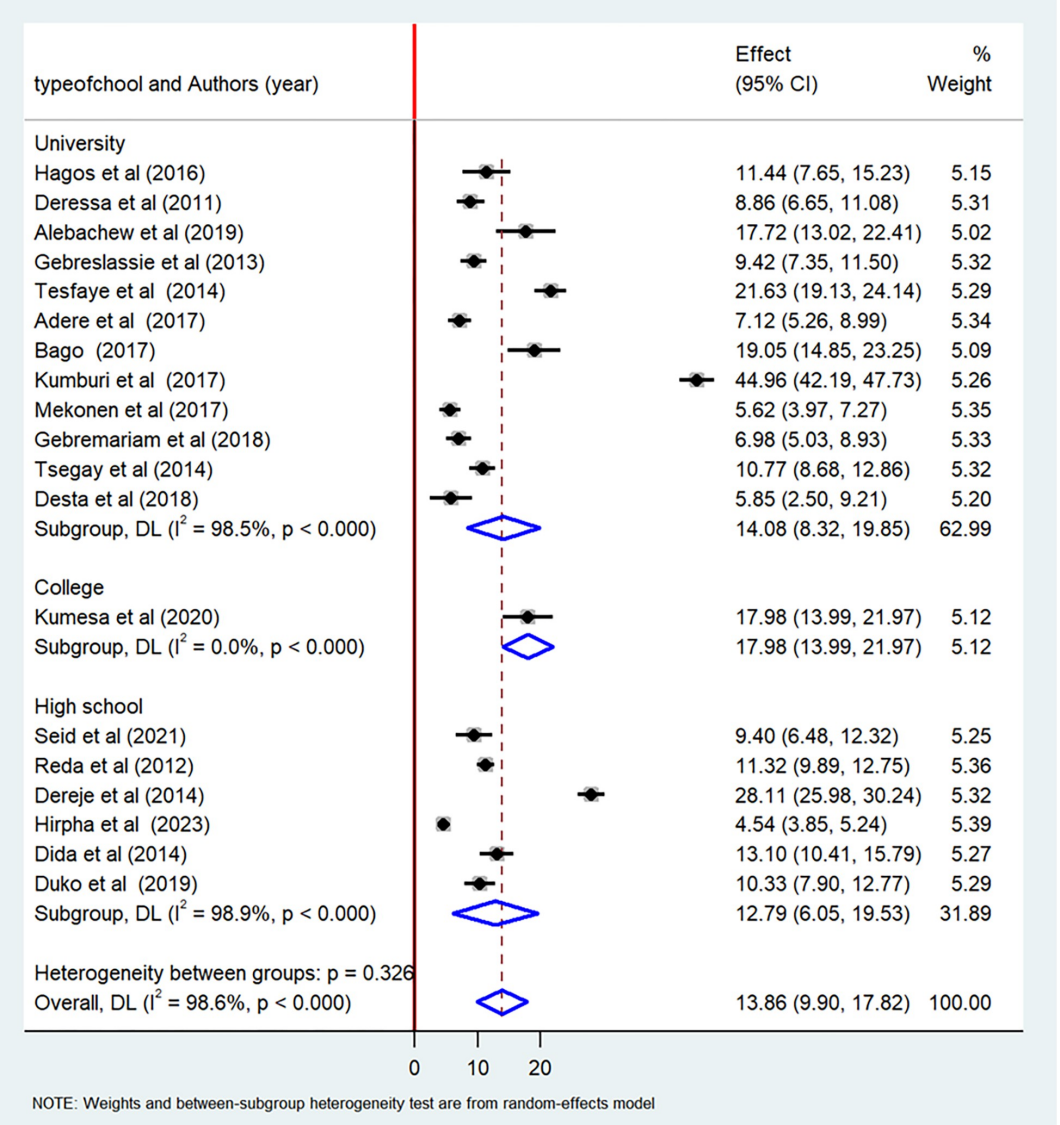
Subgroup analysis by type of school of the pooled prevalence of lifetime cigarette smoking among students in Ethiopia, 2023.

In this systematic review and meta-analysis, we conducted subgroup analyses based on the type of student (university, high school, or college) and the region where the studies were conducted. In terms of region, the pooled prevalence of lifetime cigarette smoking was found to be highest among students in the Oromia region (17.44%, 95% CI: 10.73–24.16), while the lowest pooled prevalence was observed among students in the Amhara region (8.26%, 95% CI: 5.90–10.61) (Fig 6). When categorized by type of student, the highest prevalence of lifetime cigarette smoking was reported among university students (14.08%, 95% CI: 8.32–19.85), followed by high school students (12.79%, 95% CI: 6.05–19.53) (Fig 7).

### Meta-regression and sensitivity analysis

**Fig 8:**
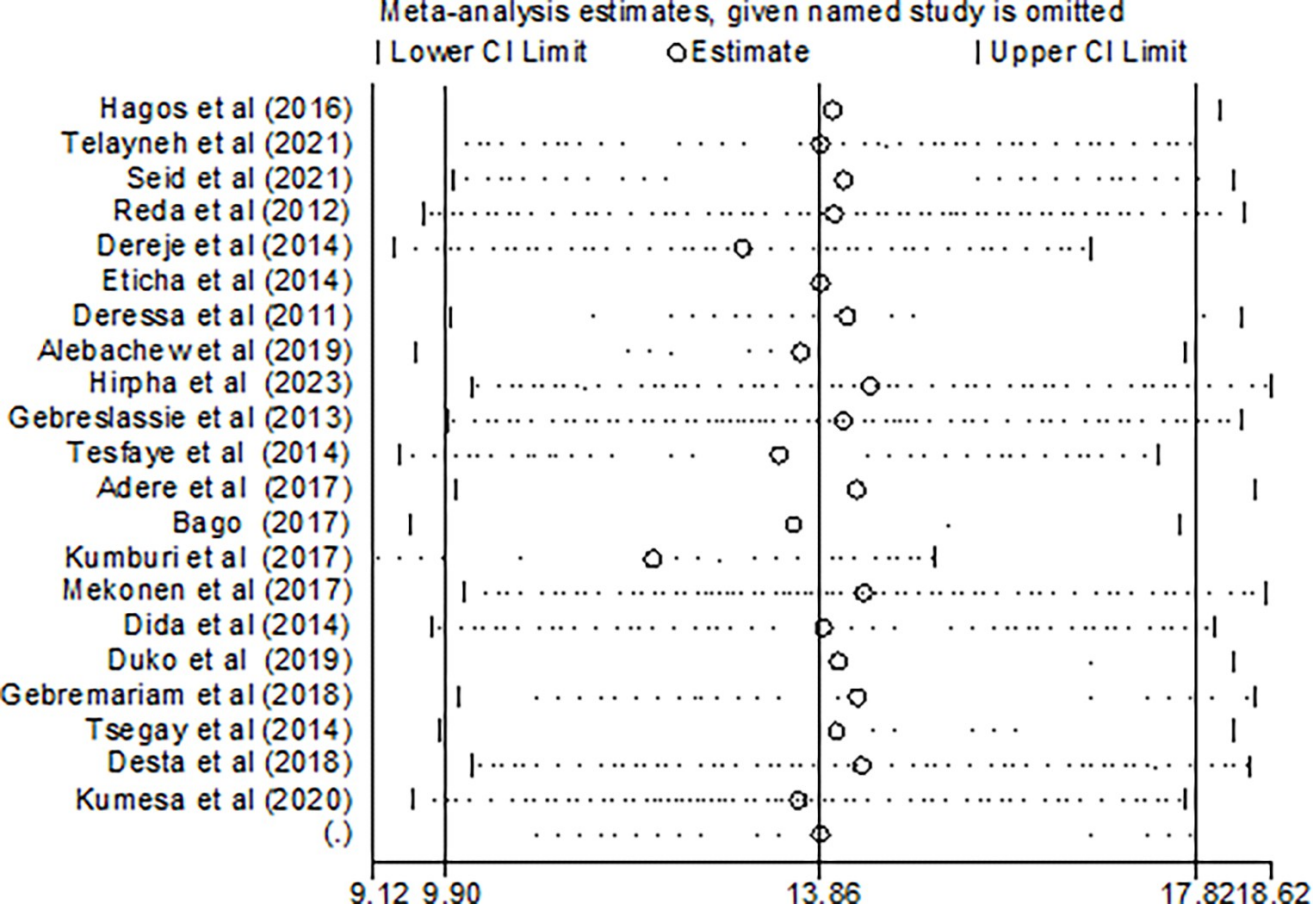
Sensitivity analysis of the included studies.

To identify the possible sources of heterogeneity, a univariate meta-regression model was conducted, considering factors, such as publication year, sample size, and type of student. However, none of these variables demonstrated statistical significance (Table 2). Furthermore, a sensitivity analysis was performed to evaluate the impact of individual studies on the overall pooled estimate of cigarette smoking, and the results indicated that no single study exerted a significant effect (Fig 8).

**Table 2:** Univariate meta-regression analysis to identify factors associated with the heterogeneity of the prevalence of cigarette smoking in Ethiopia, 2023.

Variables	Coefficient	P-value
Type of school	-.0976517	0.803
Sample size	-.7033893	0.401
Year of publication	.8179578	0.106

### Factors associated with lifetime cigarette smoking

**Fig 9:**
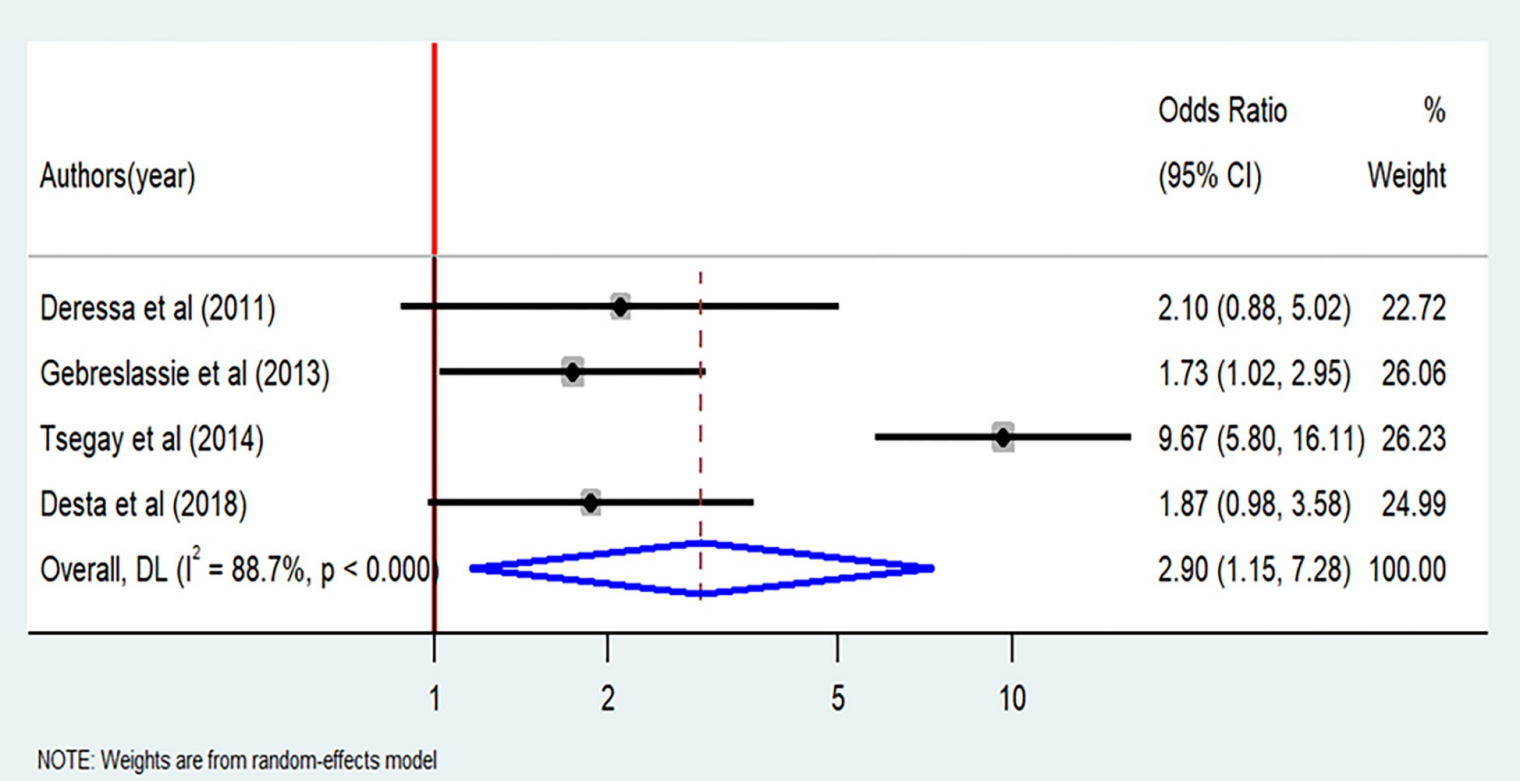
Forest plot of odds ratio for the association between residence and cigarette smoking among students in Ethiopia, 2023.

**Fig 10:**
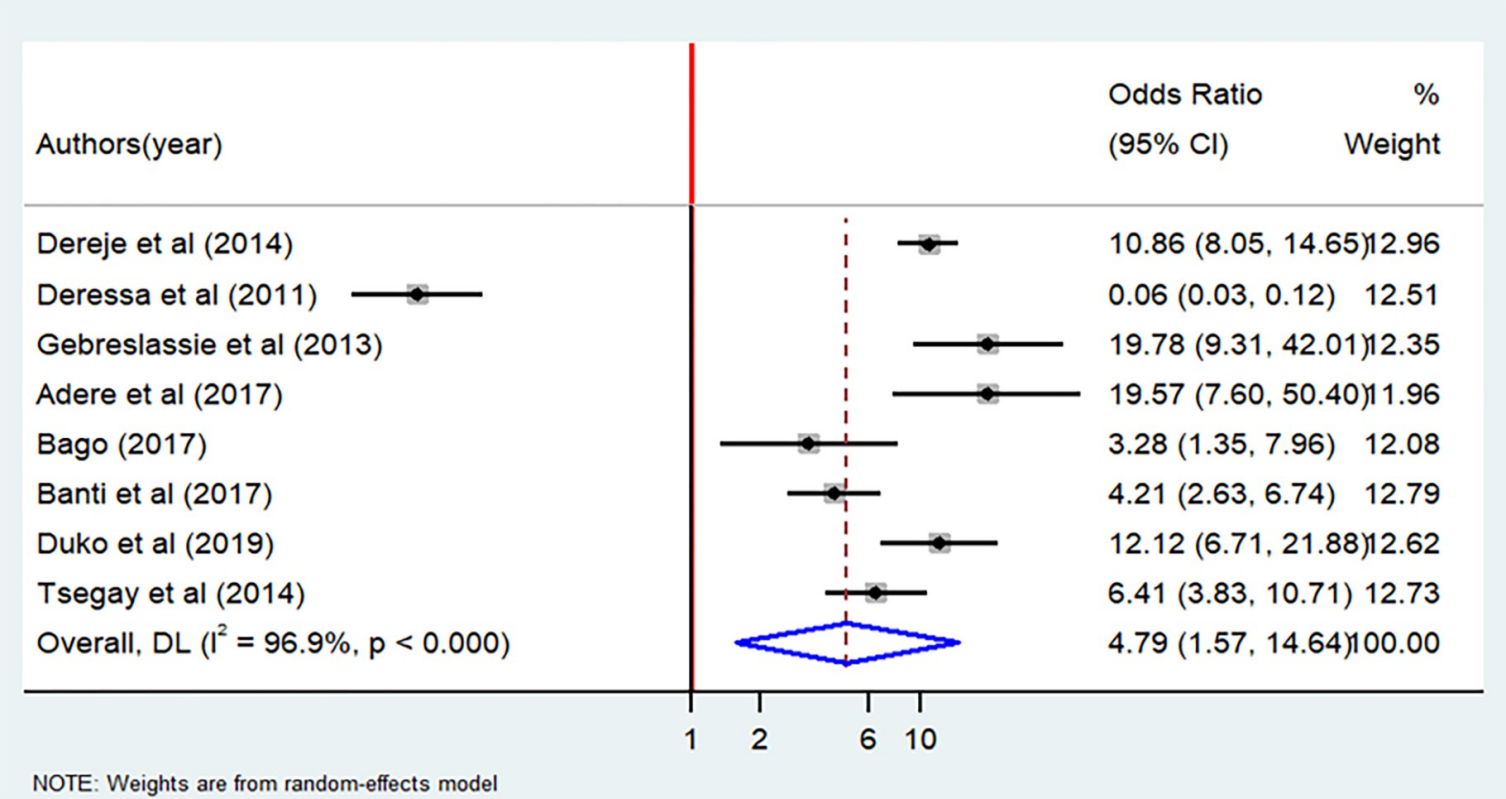
Forest plot of odds ratio for the association between alcohol consumption and cigarette smoking among students in Ethiopia, 2023.

As displayed in Figs 9 and 10, living in urban areas and alcohol consumption are significant factors associated with cigarette smoking. Urban resident students were almost three times more likely to smoke cigarettes than rural students (OR = 2.9; 95% CI: 1.15–7.28). Indeed, significant heterogeneity was observed among the included articles (I^2^ = 93.1%, P < 0.001) (Fig 9). Furthermore, the analysis revealed that students who have alcohol consumption habit had odds of cigarette smoking that were four times higher than their counterparts (OR = 4.79; 95% CI: 1.57–14.64). However, the included articles exhibited extreme heterogeneity (I^2^ = 96.9%, P <0.001) (Fig 10).The results from two studies [19, 33] indicated positive association between having health information and cigarette smoking, while three studies [22, 29, 37] suggested a negative association. However, when subjected to meta-analysis, the presence of health information about cigarette smoking did not demonstrate a significant association with cigarette smoking, with an odds ratio of 0.74 (95% CI: 0.11–5.03) (S3 File).

### The association between illegal drug use and cigarette smoking

**Fig 11:**
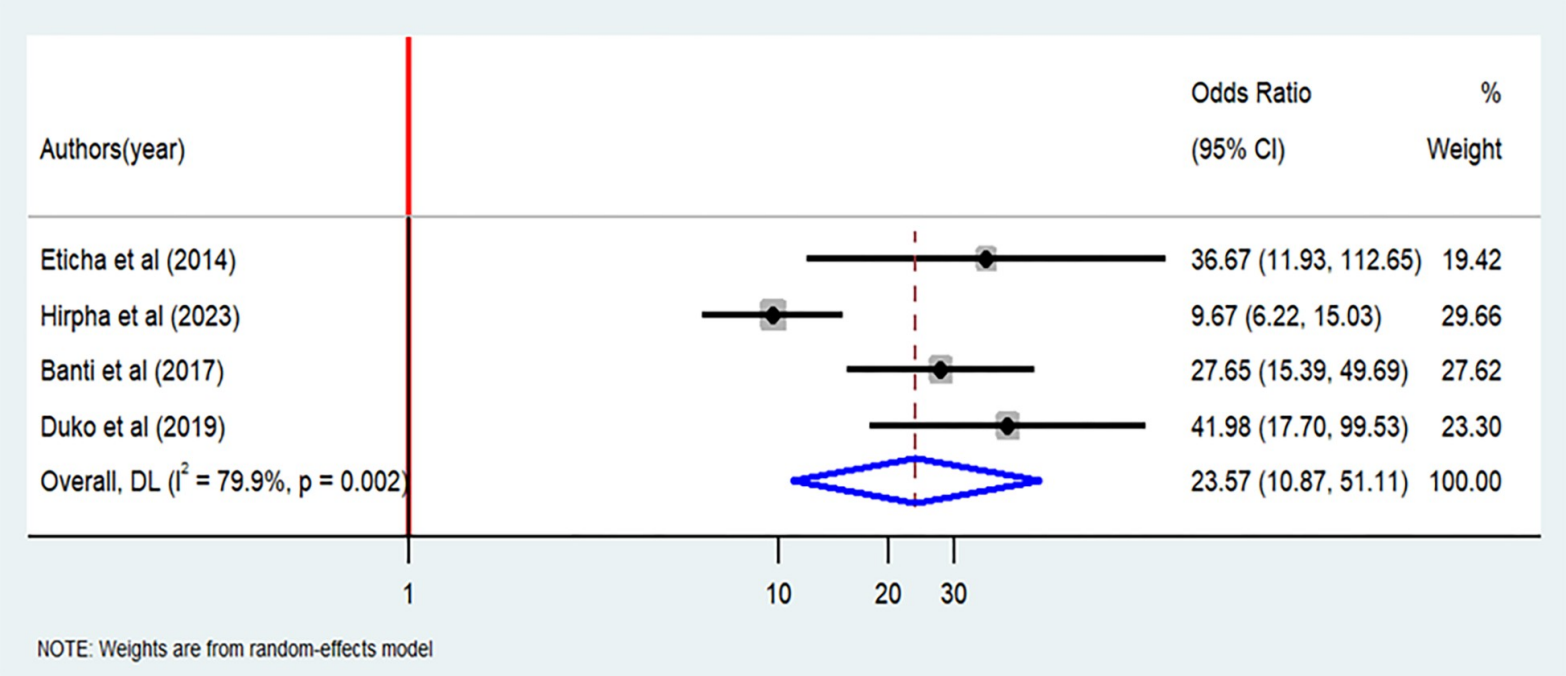
Association between illegal drug use and cigarette smoking among students in Ethiopia, 2023.

Among the twenty-two studies included, four of them [20–23] specifically examined the association between illegal drug use and cigarette smoking. All of these studies consistently demonstrated a significant association. The results from the random-effects analysis revealed notably higher odds of cigarette smoking among students who had engaged in illegal drug use compared to those who had not (OR = 23.57; 95% CI: 10.87–51.1) (Fig 11).

## Discussion

The pooled prevalence of lifetime cigarette smoking was found to be 13.8% (95% CI: 9.90–17.82). This figure is lower compared to previous reports in India (54%) [43], Kenya (38.6%) [44], Cameroon (93.1%) [45], Zimbabwe (28.8%) [46], South Africa (16.9%) [47], and Jamaica (16.7%) [48]. The low figure in Ethiopia could be associated with inaccessibility and unavailability of substances, and the influence of strict and condemning cultural and religious norms in the society.

The student-type subgroup analysis conducted in this meta-analysis revealed significant variation in cigarette smoking among different types of students. The finding indicated that university students had a higher level of cigarette smoking as compared to high school students. Our finding was consistent with a previous systematic review and meta-analysis conducted in Ethiopia [5]. This high prevalence among university students could be due to the fact that most of the university students do not live with their families, and therefore they are free from family control. As a result, the students may start a new life away from their families, which could involve cigarette smoking [49].

Our meta-analysis showed that the odds of cigarette smoking were twenty-three times higher among the students who used illegal drugs than those who did not use illegal drugs. This finding is consistent with studies conducted in Thailand [50], six European countries [51], university students in Iran [52], and college students in United States [53]. This finding is also supported by a study conducted in Malaysia, which indicated that the students who used illegal drugs were six times more likely to experience cigarette smoking than their counterparts [54]. While utilizing illegal drugs, the students could easily access cigarette as well. The students could also be easily influenced by their illegal-drug-using peers, which would enhance the prevalence of cigarette smoking. In fact, previous reports also indicated that students who had cigarette smoking friends were more likely to experience cigarette smoking than the students who had no cigarette smoking friends [5].

The students who lived in the urban residence were three times more likely to experience cigarette smoking than their counterparts, which is consistent with studies done in Sub-Saharan Africa [55] and Ethiopia [56]. Conversely, our finding contradicts with other studies done in the USA [57] and Sub-Saharan Africa [58]; which indicated that rural residents were more likely to smoke than urban residents. This might suggest misclassification of urban and rural residences in the sample can produce misleading results, which should be interpreted with caution. Of course, differences between the study countries and the level of urbanization also matters. The odds of cigarette smoking were five times higher among the students who experienced alcohol consumption as compared to the students who did not drink alcohol. This finding was consistent with studies done in Bolivia [59], Jimma (Ethiopia) [60, 61], Zimbabwe [46], and China [62]. This could be because different forms of substance abuses, such as cigarette smoking and alcohol consumption, are highly interrelated.

### Limitation and strength of the study

This systematic review and meta-analysis used an updated PRISMA checklist, ensuring a high-quality and reliable analysis for readers. To our knowledge, this meta-analysis was the first research that explored the association between illegal drug use and cigarette smoking among students in Ethiopia, which could help for policy and decision makers, and researchers. However, this meta-analysis did not represent all regions of Ethiopia as only four studies were included to examine the association between illegal drug use and cigarette smoking.

## Conclusions

More than one in eight of the Ethiopian students had experienced lifetime cigarette smoking. We found that illegal drug use has a significant association with cigarette smoking. Urban residence and alcohol use were also identified as the determinants of the student’s lifetime cigarette smoking. Therefore, the government, ministry of education, universities, and directors of schools should create awareness about the health effects of illegal drug and cigarette smoking among the students. Moreover, illegal drug use, cigarette smoking, and alcohol control law enforcement should be strengthened. As only few cross-sectional studies are included in this meta-analysis, future investigations are needed to determine the causal relationship between illegal drug use and cigarette smoking, and hence to explore effective intervention strategies.

## Supporting information

S1 Checklist PRISMA 2020 checklist. (DOCX)

S1 File (XLSX)

S2 File Results of JBI quality assessment. (DOCX)

S3 File (DOCX)
