# Supplementary material for: Association between illegal drug use and cigarette smoking among Ethiopian students: A systematic review and meta-analysis
Source: PLoS One. 2024 Jun 20;19(6):e0304948. doi: 10.1371/journal.pone.0304948 (PMC11189178; doi:10.1371/journal.pone.0304948)
Supplement: S2 File — (DOCX) [file pone.0304948.s003.docx]

**Results of JBI Quality Assessment**

| Studies | Clear eligibility criteria | Description of study subject and study setting | Valid and reliable method to measure the exposure | Standard criteria used for measurement of the condition | Identification of confounding factors | Develop of strategies to deal with confounding factors | Valid and reliable method to measured outcomes | Appropriate statistical analysis | Total score out of 8 | Level of bias (%) |
| --- | --- | --- | --- | --- | --- | --- | --- | --- | --- | --- |
| Hagos et al | Yes | Yes | Yes | Yes | No | Yes | Yes | Yes | 7 | 87.5 |
| Telayneh et al | Yes | Yes | Yes | Yes | No | No | Yes | Yes | 6 | 75.0 |
| Seid et al | Yes | Yes | Yes | Yes | No | Yes | Yes | Yes | 7 | 87.5 |
| Reda et al | Yes | Yes | Yes | Yes | No | No | Yes | Yes | 6 | 75.0 |
| Dereje et al | Yes | Yes | Yes | Yes | No | Yes | Yes | Yes | 7 | 87.5 |
| Eticha et al | Yes | Yes | Yes | Yes | No | Yes | Yes | Yes | 7 | 87.5 |
| Deressa et al | Yes | Yes | Yes | Yes | Yes | Yes | Yes | Yes | 8 | 100 |
| Alebachew et al | Yes | Yes | Yes | Yes | Yes | Yes | Yes | Yes | 8 | 100 |
| Hirpha et al | Yes | Yes | Yes | Yes | Yes | No | Yes | Yes | 7 | 87.5 |
| Gebreslassie et al | Yes | Yes | Yes | Yes | Yes | No | Yes | Yes | 7 | 87.5 |
| Gebremariam et al | Yes | Yes | Yes | Yes | No | No | Yes | Yes | 6 | 75.0 |
| Tesfaye et al | Yes | Yes | Yes | Yes | No | No | Yes | Yes | 6 | 75.0 |
| Adere et al | Yes | Yes | Yes | Yes | No | Yes | Yes | Yes | 7 | 87.5 |
| Bago et al | Yes | Yes | Yes | Yes | Yes | Yes | Yes | Yes | 8 | 100 |
| Kumesa et al | Yes | Yes | Yes | Yes | No | Yes | Yes | Yes | 7 | 100 |
| Banti et al | Yes | Yes | Yes | Yes | No | Yes | Yes | Yes | 7 | 87.5 |
| Tsegay et al | Yes | Yes | Yes | Yes | No | No | Yes | Yes | 6 | 75.0 |
| Kumburi et al | Yes | Yes | Yes | Yes | No | No | Yes | Yes | 6 | 75.0 |
| Mekonen et al | Yes | Yes | Yes | Yes | No | Yes | Yes | Yes | 7 | 87.5 |
| Dida et al | Yes | Yes | Yes | Yes | No | Yes | Yes | Yes | 7 | 87.5 |
| Desta et al | Yes | Yes | Yes | Yes | No | Yes | Yes | Yes | 7 | 87.5 |
| Duko et al | Yes | Yes | Yes | Yes | Yes | Yes | Yes | Yes | 8 | 100 |
