# Supplementary material for: Association between illegal drug use and cigarette smoking among Ethiopian students: A systematic review and meta-analysis
Source: PLoS One. 2024 Jun 20;19(6):e0304948. doi: 10.1371/journal.pone.0304948 (PMC11189178; doi:10.1371/journal.pone.0304948)
Supplement: S3 File — (DOCX) [file pone.0304948.s004.docx]

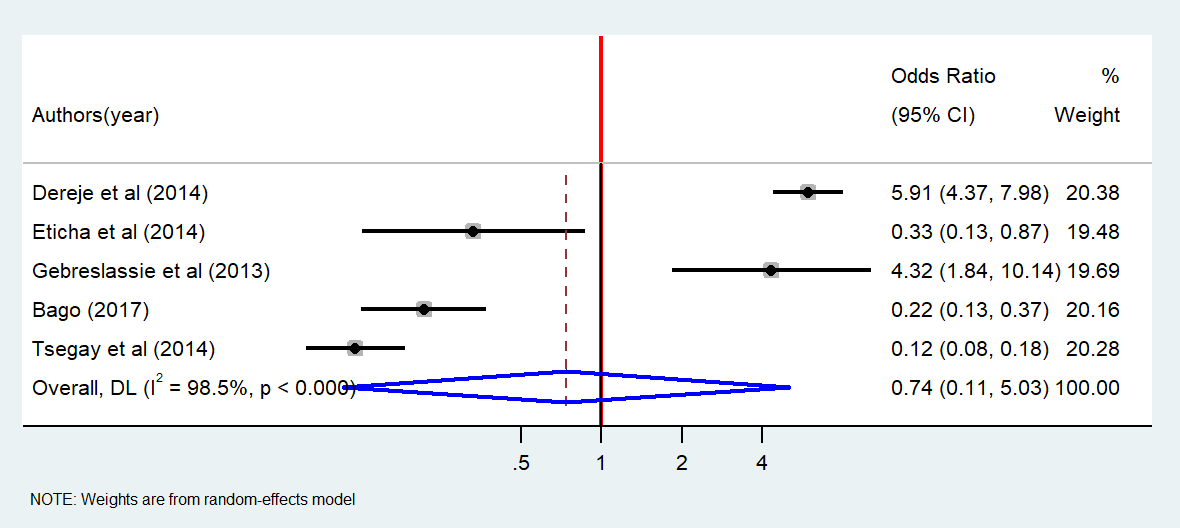


Figure 1: Forest plot of odds ratio for the association between having health information and cigarette smoking among students in Ethiopia, 2023.
